# Supplementary material for: Causal Relationship between Aging and Anorexia Nervosa: A White-Matter-Microstructure-Mediated Mendelian Randomization Analysis
Source: Biomedicines. 2024 Aug 16;12(8):1874. doi: 10.3390/biomedicines12081874 (PMC11351342; doi:10.3390/biomedicines12081874)
Supplement: Supplementary file 1 [file biomedicines-12-01874-s001.zip › Supplementary Figure/Figure S7/Single SNP plot of MR analysis for ubm-b-1939 to AN.pdf]

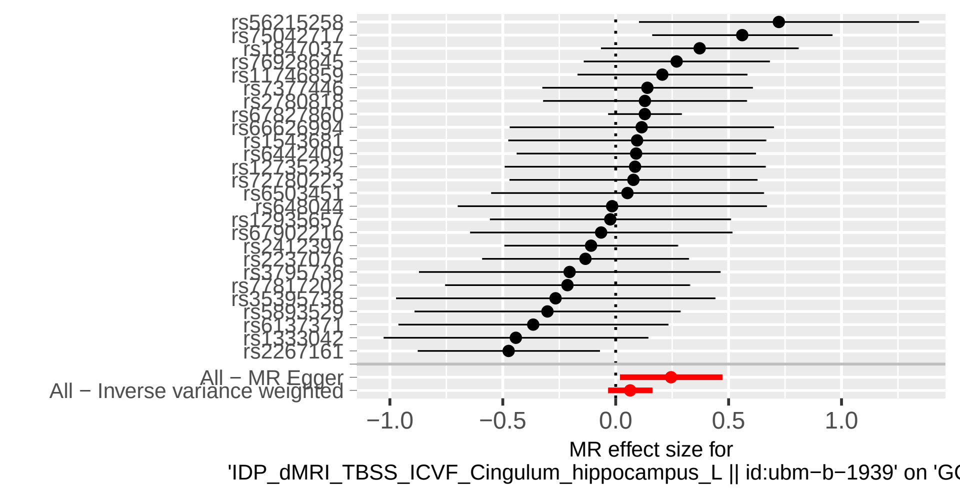
rs56215258
rs75042717
rs1847037
rs76928645
rs11746859
rs7377446
rs2780818
rs67827860
rs66626994
rs1543681
rs6442409
rs12735232
rs72780223
rs6503451
rs648044
rs12935657
rs67902216
rs2412397
rs2237076
rs3795736
rs77817202
rs35395738
rs5893529
rs6137371
rs1333042
rs2267161
All − MR Egger
All − Inverse variance weighted
−1.0
−0.5
0.0
0.5
1.0
MR effect size for
'IDP_dMRI_TBSS_ICVF_Cingulum_hippocampus_L || id:ubm−b−1939' on 'G
